# Patients with non-operated traumatic primary or recurrent anterior shoulder dislocation have equally poor self-reported and measured shoulder function: a cross-sectional study

**DOI:** 10.1186/s12891-019-2444-0

**Published:** 2019-02-08

**Authors:** Henrik Eshoj, Sten Rasmussen, Lars Henrik Frich, Steen Lund Jensen, Karen Søgaard, Birgit Juul-Kristensen

**Affiliations:** 1 Quality of Life Research Center, Department of Hematology, Odense University Hospital, J. B. Winsløws Vej 4, DK-5000 Odense, Denmark; 2 Orthopaedic Research Unit, Aalborg University Hospital, Aalborg, Denmark, Hobrovej 18-22, DK-9000 Aalborg, Denmark; 3 Department of Clinical Medicine, Aalborg University, Hobrovej 18-22, DK-9000 Aalborg, Denmark; 4 Department of Orthopaedics and Traumatology, Odense University Hospital, J. B. Winsløws Vej 4, DK-5000 Odense, Denmark; 5 Department of Clinical Research, University of Southern Denmark, Campusvej 55, DK-5230 Odense, Denmark; 6 Shoulder sector, Orthopaedic Department, Aalborg University Hospital, Farsoe, Denmark; 7 Department of Sports Science and Clinical Biomechanics, University of Southern, Campusvej 55, DK-5230 Odense, Denmark

**Keywords:** Shoulder dislocation, shoulder instability, Re-injury, Proprioception, Range of movement, Strength, Kinesiophobia, Quality of life, Males

## Abstract

**Background:**

Patients with non-operated traumatic primary anterior shoulder dislocation (PASD) are assumed to have less shoulder impairment than patients with recurrent anterior shoulder dislocations (RASD). This may impact treatment decision strategy. The aim was to study whether patients with non-operated traumatic PASD have less shoulder impairment than those with RASD.

**Methods:**

In a cross-sectional study baseline data from patients with PASD and RASD in a randomised controlled trial of non-operative shoulder exercise treatment were used. Shoulder function was self-reported (Western Ontario Shoulder Instability (WOSI), Tampa Scale of Kinesiophobia (TSK), General Health (EQ-5D-VAS), Numeric Pain Rating Scale (NPRS)), and measured (Constant-Murley shoulder Score (CMS total), CMS - Range of Motion (CMS-ROM, CMS – strength, proprioception, clinical tests).

**Results:**

In total, 56 patients (34 (28 men) with PASD and 22 (21 men) with RASD) (mean age 26 years) participated. WOSI total was 1064 and 1048, and TSK above 37 (indicating high re-injury fear) was present in 33 (97%) and 21 (96%) of the groups with PASD and RASD, with no group difference. CMS total (66.4 and 70.4), CMS-ROM (28.7 and 31.5), CMS-strength (injured shoulder: 7.6 kg and 9.1 kg), proprioception and clinical tests were the same. Furthermore, 26 (76%) with PASD and 13 (59%) with RASD reported not to have received non-operative shoulder treatment.

**Conclusions:**

Non-operated patients with PASD and self-reported shoulder trouble three-six weeks after initial injury do not have less shoulder impairment (self-reportedly or objectively measured) than non-operated patients RASD and self-reported shoulder trouble three-six weeks after their latest shoulder dislocation event.

## Background

Traumatic primary anterior shoulder dislocation (PASD) is frequent in active athletic individuals, which may have negative consequences for shoulder function, physically as well as mentally [1–3]. Following traumatic PASD there is, subsequently, a higher risk (39%) of experiencing recurrent anterior shoulder dislocation (RASD) [4]. Risk factors associated with RASD are age (below the age of 40), male gender, generalized joint hypermobility (GJH), and shoulder laxity, besides a history of fractures to the greater tuberosity of the humerus [4, 5].

With RASD, deficits in the global neuromuscular [6] and proprioceptive systems [7, 8] in addition to weakness of the rotator cuff muscles [9] often follow, thereby impairing shoulder stability and control. Presumably, this may also affect shoulder-related quality of life and possibilities for sport participation [10]. Actually, it is assumed that for every repeated anterior shoulder dislocation, shoulder function will deteriorate, thereby impairing shoulder joint stability in a vicious circle leading to complex shoulder impairment (physically as well as mentally) [11]. Thus, patients with RASD may, to a higher degree than patients with PASD, develop worse shoulder impairments, leading to the anticipation that those with PASD have less indication for shoulder treatment. In general, this is also how current practice [12, 13] is described today with most surgeons taking on a “wait and see” treatment approach in relation to immediate operation in patients with traumatic PASD [14]. Unfortunately though, taking the high risk of RASD following a PASD into account, a Dutch survey on the management of patients with PASD revealed that only 60% of the orthopaedic surgeons at public hospitals in the Netherlands routinely refer non-operatively treated PASD patients to physical therapy [15]. This seems not to be the ideal prevention strategy to avoid RASD. One reason, however, may be that the evidence, for prescribing such supervised post-traumatic rehabilitation for PASD patients is highly lacking [16, 17]. Another reason may be, that patients with traumatic PASD are anticipated to have less shoulder impairment than patients with RASD, which may justify the “wait and see” approach for PASD. However, support for the latter statement is still unknown and therefore investigated in the present study. Therefore, the purpose of this study was to investigate whether patients with traumatic PASD have less shoulder impairment than patients with RASD.

## Methods

This cross-sectional study draws on baseline data from patients with traumatic PASD and RASD included in a randomised controlled multi-centre trial (The SINEX study) [18] investigating the effect of a non-operative neuromuscular shoulder exercise program. Patients were recruited 3–6 weeks (sub-acute phase) after their latest anterior shoulder dislocation from shoulder outpatient clinics of orthopaedic departments in the Region of Northern Denmark (Aalborg University Hospital, Aalborg) and Southern Denmark (Odense University Hospital, Odense and South-West Jutland Hospital, Esbjerg). The trial is described elsewhere [18] and registered at the National Institutes of Health Clinical Trials Protocol Registration System: ClinicalTrials.gov identifier (NCT02371928).

### Population

In total, 56 patients were included in the aforementioned SINEX trial and therefore it also constitutes the current study population. The eligibility criteria for the SINEX study were men and women aged 18–39 years with a traumatic anterior shoulder dislocation (primary or recurrent event, with a maximum of up to five anterior dislocations). Furthermore, patients in the SINEX study needed to meet the following inclusion criteria: a minimum of one radiological verified anterior shoulder dislocation, in addition to self-reported shoulder trouble in the week prior to assessment for trial inclusion, e.g. reduced ability to perform specific shoulder movements during sports/leisure activity and/or work. Exclusion criteria for the SINEX study were the following: humeral fracture and/or bony Bankart lesion warranting surgery, prior surgery in the affected shoulder joint, more than five anterior shoulder dislocations (verified by patient register or subjective evaluation), suspected competing diagnosis (e.g. rheumatoid arthritis, cancer, neurological disorders, fibromyalgia, schizophrenia, suicidal tendency, borderline personality disorder or obsessive compulsive disorder), sensory and motor deficits in neck and shoulder, pregnancy, inadequacy to write and speak Danish, and unwillingness or inability to attend 12 weeks of a physical therapist-supervised neuromuscular shoulder exercise program.

### Data collection procedures

Patient reported outcomes were completed electronically to avoid influence from clinical outcome assessors and risk of data entry errors, and for the objective data, two clinical outcome assessors (both physical therapists) performed all data collection. Thoroughly described test protocols were used and, before data collection, the two clinical outcome assessors trained together to align their testing and interpretation procedures.

### Outcome variables

#### Self-reported outcomes

The primary outcome was the Western Ontario Shoulder Instability Index (WOSI, 0–2100, 0 = no trouble) questionnaire [19], recently translated into Danish with satisfactory reliability and validity in a Danish population with shoulder instability [20]. For assessment of shoulder-related function and Quality of Life (QoL), the total WOSI score in addition to the individual domains of WOSI were calculated, covering ‘Physical symptoms’ (0–1000), ‘Sport/recreation/work’ (0–400), ‘Life-style’ (0–400) and ‘Emotions’ (0–300) [19].

Fear of movement and re-injury was measured by the Tampa Scale of Kinesiophobia (TSK) questionnaire, ranging from 17 to 68 (68 representing worst score), with 17 items each using a four-point Likert scale. TSK scores above 37 represent high fear of re-injury [21]. TSK has been translated to Danish following general scientific principles and validated in a Danish population of workers with different levels of pain and various musculoskeletal complaints [22]. Also, the TSK has been used as treatment effect measures in a number of Danish studies [23–25] and has satisfactory psychometric properties in a population similar to the current primary care patients with shoulder pain, although in an American setting [26].

For health-related QoL, the Visual Analogue Scale (EQ-VAS) from the Danish version of the EQ-5D was used, ranging from 0 to 100 (0 = lowest score) [27, 28], validated and recommended as instrument for measures of health state and economic analysis [29]. Finally, pain intensity during the previous 24 h was reported using the Numeric Pain Rating Scale (NPRS), ranging from 0 to 10 (10 = worst imaginable pain) [30]. NPRS has shown to have satisfactory psychometric properties in a population with shoulder pain [31].

#### Clinical outcomes

##### Generalized joint hypermobility

Patients were tested for generalized joint hypermobility (GJH) with the use of Beighton’s criteria consisting of nine tests (positive/negative, ranging from 0 to 9), where ≥4 was classified as GJH [32]. Beighton tests have been shown to be reliable for measuring GJH [33]. Briefly, the tests include: forward bending while standing with legs straight placing both palms relaxed on the ground (one point), bilateral elbow hyperextension above 10 degrees (one point per side), bilateral knee hyperextension above 10 degrees (one point per side), bilateral fifth finger extension backwards above 90 degrees (one point per side) and bilateral thumb in apposition and touching the flexor side of the forearm (one point per side).

##### Anterior shoulder instability

Clinical anterior shoulder instability was evaluated by the three instability and pain-provocative tests of apprehension, relocation and surprise [34], recently shown to be reliable in patients with and without self-reported shoulder instability and laxity [35]. Briefly, patients were placed in supine lying with the shoulder being tested placed close to the edge of the examination table.

For the apprehension test, the elbow was in 90 degrees of flexion, and the shoulder in 90 degrees of abduction. The shoulder was then externally rotated into maximal end range. The test was considered positive if this movement produced apprehension and/or pain located on the anterior side of the glenohumeral joint.

The relocation test was performed immediately after the apprehension test by gently performing a posteriorly directed force to the anterior side of the humeral head. The relocation test was considered positive if there was relief of apprehension and/or pain after humeral head relocation.

To execute the surprise test, the posteriorly directed force performed in the relocation test was suddenly released. The surprise test was considered positive if apprehensive signs and/or pain returned following removal of the posteriorly directed force to the humeral head.

If any of the clinical tests could not be performed due to limited shoulder range of motion, pain or anxiety, those tests were likewise considered positive.

#### Measured functional outcomes

##### The constant-Murley shoulder score (CMS)

The CMS was used to evaluate subjective and objective shoulder function via patient-reported questions and functional shoulder tests (Range of Motion (ROM) and strength). Total CMS is scored from 0 to 100, with 100 being the best possible shoulder function [36]. CMS has reached satisfactory reliability and validity for a variety of shoulder pathologies [37]. Further, the CMS subscales for assessing ROM (CMS-ROM) and strength (CMS-strength) were individually reported. CMS-ROM was evaluated for flexion, abduction, internal and external rotation and rated according to the CMS protocol (0–40; 40 = best). CMS-strength was tested with subjects standing evenly on 2 feet with 90 degrees of shoulder abduction in the scapular plane performing three isometric Maximal Voluntary Contractions (iMVC) in random order for each shoulder (injured and non-injured) using an Isoforce Dynamometer. If the third or last iMVC was more than 5% larger than either of the previous two, the test was repeated up to five times. The highest iMVC value for each shoulder was used for further analysis.

##### Shoulder joint position sense

To measure shoulder proprioception, a Joint Position Sense (JPS) test was used, as it has been shown to have satisfactory reliability for measuring shoulder JPS flexion and abduction (up to 60 ± 10 degrees) [38]. Briefly, a laser pointer was attached just above the elbow joint pointing towards a vertical target scale (centimetre) attached on the wall. While standing in front of the scale, patients flexed their arm to 90 degrees of shoulder flexion (thumb pointing upwards). In this position, the target scale and laser pointer were adjusted so that the laser dot pointed to the center of the target scale. This position was scored 0 points and was the reference point for the following JPS measurements. Before the actual testing, patients were positioned at a distance equal to one arm’s length + 10 cm from the target scale (arm length = length from the anterior part of the acromion to the tip of the third finger, with the arm positioned in 90 degrees of shoulder flexion). The test was performed in shoulder flexion (the front of the body pointing towards the target scale), as well as in abduction (the side of the body pointing towards the target scale), with the same instructions for performing all JPS tests. For shoulder flexion, the JPS test was performed as follows: Patients were blindfolded, standing evenly on 2 feet, with arms resting alongside the body. Patients were pre-instructed to flex their shoulder (thumbs pointing upwards) and stop on a verbal instruction by the experimenter when the laser dot fell within a predefined range equal to 60 ± 10 degrees of shoulder flexion (position pre-calculated by use of trigonometry and Pythagoras´ Theorem and marked on the target scale). Patients were then told to bring back their arm to neutral and immediately afterwards to reproduce the shoulder position as before. Patients were instructed to tell the experimenter when they perceived that the previous position was reached. The two positions (the reference and reposition point) were marked to calculate the difference in centimetre from the reference point to the repositioned point, thereby representing the shoulder JPS error.

Flexion and abduction tests were repeated three times each and only the test with the median score was used for analysis.

#### Demographics and historical information

Demographic data included the following: sex, age, weight, height, educational level, employment status, dominant arm (right/left) and injured shoulder (right/left).

Historical information included: injury mechanism, total number of shoulder reductions treated in an orthopaedic unit (1–5), prior shoulder treatment (no/yes), and if yes, which treatment. Lastly, patients were asked whether they were physically active before their latest shoulder dislocation injury (no/yes), and if yes, at what level of physical activity (hours/week).

### Statistical methods

Data were checked for normality using the Shapiro Wilk’s test, visual inspection of histograms and QQ plots. Descriptive analyses are presented as frequencies (%) or mean values with standard deviations (SD).

To test for group differences between PASD and RASD, independent t-tests were used for normally distributed data; for non-normally distributed continuous data, the Mann-Whitney U-test was used. For categorical outcomes (clinical tests), the Fischer’s Exact test was chosen. Since the current study population constitutes baseline data from an RCT [18], no power calculation was performed specifically for the current study. The level of significance was defined as *p* < 0.05. To adjust for multiple comparisons, a Bonferroni correction was used where *p*-values were less than 0.05. All statistical analyses were performed with the Statistical Package for the Social Sciences (SPSS, IBM, Armonk NY, USA), version 24.0.

## Results

A total of 56 patients with traumatic anterior shoulder dislocation were included in the study (primary: *n* = 34; recurrent: *n* = 22). The groups were similar regarding demographics (Table 1), with a mean age of 26 years (SD 6) and a majority being males (82% (*n* = 28) and 96% (*n* = 21), respectively). In most cases, the injured arm was the dominant arm and the injury was primarily due to ‘a fall on the arm’, and both groups (88% (*n* = 30) and 68% (*n* = 15)) had been physically active prior to their recent anterior shoulder dislocation event. In total, 76% (*n* = 26) and 59% (*n* = 13) from the patients with PASD and RASD, respectively, reported that they had not received any shoulder treatment following their primary anterior shoulder dislocation (Table 1).

**Table 1:** Demographic and historical information in patients with traumatic primary (PASD) or recurrent anterior shoulder dislocation (RASD)

Variables	PASD *n* = 34)	RASD (*n* = 22)
Sex (male (%))	28 (82)	21 (96)
Age (years) Mean (SD)	26 (7)	25 (5)
Weight (kg) Mean (SD)	84.0 (19.8)^a^	82.4 (15.8)
Height (cm) Mean (SD)	178 (7.6)^b^	181 (8.6)
Occupation (n (%))		
Academic education	12 (35)	5 (23)
Skilled	12 (35)	12 (54)
Unskilled	5 (15)	2 (9)
No education	5 (15)	3 (14)
Employment status (n (%))		
Full-time	14 (41)	16 (73)
Part-time	2 (6)	–
Student	15 (44)	4 (18)
Unemployed/retired	–	–
Sick listed	3 (9)	2 (9)
Dominant arm (right (%))	30 (88)	21 (96)
Injured shoulder (right (%))	15 (44)	13 (59)
Injury mechanism (n (%))		
Fall on the arm	17 (50)	11 (50)
Distraction of the arm	6 (18)	1 (5)
External force to the shoulder	2 (6)	1 (5)
Other	9 (26)	9 (40)
Number of shoulder reductions treated in an orthopaedic unit (n (%))		
Unknown	–	4 (18)
1	34 (100)	–
2	–	9 (41)
3	–	5 (23)
4	–	3 (14)
5	–	1 (4)
Have you previously received any shoulder treatment? (n (%))		
No	26 (76)	13 (59)
Yes	7 (21)	9 (41)
Active PT exercise treatment	6 (86)	7 (78)
Passive treatment	3 (43)	3 (33)
Chiropractic	1 (14)	–
Analgesic medication (medically prescribed)	3 (43)	3 (33)
Are you physically active? (n (%))		
No	4 (12)	7 (32)
Yes	30 (88)	15 (68)
≥ 4 h/week	25 (83)	13 (86)

*SD* Standard deviation, ^a^Missing data = 2; ^b^Missing data = 1

There was no group difference in self-reported shoulder function (Table 2), and shoulder-related QoL (WOSI) was 1064.0 and 1048.3 in the PASD and RASD group, respectively, corresponding to a self-reported shoulder function of less than 50% of a healthy shoulder function in both groups. The WOSI sub-scales of Lifestyle and Emotions were mostly affected (both groups), thereby leaving only about 30 and 43% of a healthy shoulder condition. The frequency of patients with TSK scores above 37 (indicating high fear of re-injury) was 97% (*n* = 33) and 96% (*n* = 21).

**Table 2:** Self-reported and measured shoulder function in patients with traumatic primary (PASD) or recurrent anterior shoulder dislocation (RASD)

Outcomes	PASD (*n* = 34)	RASD (*n* = 22)	*P*-value
Patient-reported (Mean, SD)			
WOSI total (0–2100)	1064.0 (373.2)	1048.3 (371.5)	0.88
Physical symptoms (0–1000)	374.1 (183.5)	387.2 (191.2)	0.87
Sport function (0–400)	239.5 (101.5)	230.7 (73.1)	0.73
Lifestyle (0–400)	236.7 (85.7)	220.9 (97.7)	0.53
Emotions (0–300)	213.6 (67.5)	209.5 (63.8)	0.71
TSK (17–68)	42.5 (4.5)	44.3 (4.6)	0.12
≥ 37 (high re-injury fear), yes (%)	33 (97)	21 (96)	0.20
EQ VAS (0–100)	72.6 (17.2)	77.8 (16.5)	0.23
NPRS (Latest 24 h) (0–10)	3.4 (2.1)	3.1 (2.2)	0.52
Clinical tests			
GJH (0–9, positive ≥4, yes (%))	1 (3)	7 (31)	0.59
Clinical tests (positive, yes, (%))			
Apprehension	34 (100)	20 (96)^a^	0.38
Relocation	31 (91)	15 (71)^a^	0.07
Surprise	28 (82)	17 (81)^a^	1.00
Performance-based (Mean, SD)			
Total CMS (combined score, 0–100)	66.4 (18.1)	70.4 (19.4)	0.41
CMS-ROM (combined score, 0–40)	28.7 (8.6)	31.5 (8.7)	0.16
CMS-strength (kg)			
Injured shoulder	7.6 (3.1)	9.1 (4.0)^b^	0.24
Non-injured shoulder	11.9 (3.4)	11.7 (3.9)^b^	0.56
Shoulder JPS^a^ (cm, AE)			
Flexion	5.7 (7.8)	6.1 (6.9) ^b^	0.85
Abduction	6.5 (6.8)	7.4 (6.5) ^b^	0.67

*SD* Standard deviation, *WOSI* Western Ontario Shoulder Instability Index, *TSK* Tampa Scale of Kinesiophobia, *EQ VAS* Euroqol Visual Analogue Scale, *NPRS* Numeric Pain Rating Scale, *GJH* Generalized Joint Hypermobility, *CMS* Constant-Murley Score, *ROM* Range of Motion, *JPS* Joint Position Sense, *cm* centimetre, *AE* absolute error ^a^ Missing data (see text for further information); ^a^ Missing data = 1, ^b^ Missing data = 2

There was no group difference in the measured clinical tests, however, a larger frequency of patients with GJH was present in the RASD group corresponding to 31% (*n* = 7) versus 3% (*n* = 1) in the PASD group (Table 2). In five patients (three PASD and two RASD) the clinical anterior shoulder instability tests could not be performed due to shoulder pain, anxiety or limited range of shoulder movement. Most of the patients in both groups (71–100%) had positive clinical tests for anterior shoulder instability, with the apprehension test being the most prevalent.

Two participants in the RASD group were excluded from the CMS-strength (injured shoulder) analyses due to equipment failure. For all measured performance-based outcomes of shoulder function, no group differences were found (Table 2).

Two patients in the PASD group were not able to perform the CMS-strength test due to either shoulder pain and/or limited range of shoulder movement (90 degrees of shoulder abduction was required) and they therefore scored 0 points (according to the standardised CMS-strength protocol). Total CMS scores were 64.4 (SD 18.1) and 70.4 (SD 19.4) for the PASD versus RASD group, respectively. Finally, CMS-strength for the injured shoulder was 7.6 (SD 3.1) and 9.1 kg (SD 4.0) for the PASD and RASD group, respectively, corresponding to only 70% strength of the non-injured shoulder.

## Discussion

Patients with non-operated traumatic PASD do not have less shoulder impairment (self-reportedly or objectively measured) than patients with RASD. Further, both groups present with equally poor shoulder function and high fear of re-injury. Despite the shoulder impairments, a large proportion of this patient group (primary and recurrent anterior shoulder dislocation) had not been offered supervised shoulder rehabilitation following any of their shoulder dislocation events.

The present poor WOSI scores and general health (EQ VAS) were in line with previous studies of patients with PASD [39, 40] and RASD [41]. The present patients had a mean TSK score of 43.4 and almost all patients reported high fear of re-injury with TSK score above 37 [21], indicating a large impact on fear-related physiological and emotional conditions in both patient groups. These findings correspond well with the domains of Lifestyle and Emotion in WOSI, showing that only approximately 30 and 43% of the maximal obtainable score for healthy shoulders was present in the injured shoulder. With no group differences, these findings show that self-reported shoulder function is seriously impaired in both groups with traumatic PASD and RASD.

Concerning clinical characteristics of the current patients, GJH was present in only one of 34 (3%) of the patients with traumatic PASD as opposed to seven of 22 (33%) in the RASD group, however, with no group difference. For the total group, the prevalence of patients with positive GJH of 14% (8 of 56 patients) is in line with a previous study on patients with traumatic PASD [42], but smaller than found in a study of patients with RASD where the prevalence of GJH was four times higher [43]. However, the latter study used slightly different criteria to determine GJH, which may limit direct comparison. Nevertheless, GJH is generally reported as a risk factor for PASD and especially the subsequent experience of RASD [4, 44]. The relatively small sample size in the current study may explain the lack of group difference in GJH prevalence. The clinical tests for anterior shoulder instability were largely positive in both groups (71–100% of the present total 56 patients), which is in contrast to a previous study showing that only 46% (of 26 patients with RASD) had a positive apprehension test [43]. The explanation for this difference may rely on the small sample size and discrepancy in performance and interpretation of the apprehension test, compared with the previous study [43]. Further, using pain as a criterion for a positive test (as in the present study), which may be controversial, since pain has shown less predictive and reliable as a diagnostic criterion, may have increased the prevalence compared with using only apprehension as criterion [45, 46]. Nevertheless, the large prevalence of positive apprehension and/or pain during testing (34 of 34 (100%) and 20 of 21 (96%)) may indicate signs of joint arthropathy, possibly leading to decreased shoulder function. Further, since the number of positive tests was evenly distributed between pain and apprehension in the two groups of totally 56 patients (data not shown), pain may be as important as apprehension when examining patients for traumatic anterior shoulder dislocation. However, since no gold standard for diagnosing anterior shoulder instability exists today studies on the validity of such tests are needed.

In relation to the total CMS, no group difference was found. Surprisingly, the current mean total CMS for both groups was only 68 of the maximum obtainable score (100 being best), which is considerably poorer than healthy controls that range from 90 to 98 [47]. On the contrary, the current total CMS was markedly better than in patients with shoulder impingement (the current score 68 vs. 45) [48]. However, this may reflect some of the limited appropriateness of using total CMS (sum score) in shoulder instability patients. The reason is that CMS does not include items that directly assess shoulder instability with reduced possibilities for reporting lack of confidence in shoulder function and presences of fluctuating shoulder symptoms [49], despite the fact that CMS is frequently used on orthopedic shoulder patients (e.g. shoulder instability patients) [50]. The sub-score CMS-ROM may also be interpreted with caution since it is assessed during pain-free movements only. Hence, the current CMS-ROM test does not test total ROM, possibly explaining why the groups scored only 75% of the full ROM score.

With regard to maximum strength, no group difference between PASD and RASD was found for mean CMS-strength of neither the injured and non-injured shoulder. Further, since anterior shoulder instability is often perceived during sub-maximal tasks in this patient group [40], iMVC tests of shoulder abduction, as measured in CMS-strength, may not reflect all aspects of shoulder strength, e.g. whether muscle strength affects the risk for symptomatic anterior shoulder instability.

Lastly, the present findings on shoulder JPS were in line with a previous study on healthy subjects [51], indicating that JPS (low range shoulder flexion and abduction) seems not to be seriously affected by anterior shoulder dislocations in the present population. Since the current JPS test in low range was selected due to its satisfactory reliability, it remains unknown whether shoulder JPS in high range shoulder flexion and abduction is affected by PASD or RASD [35].

The fact that a large proportion of the present patients reported not to have been offered any supervised rehabilitation (such as physical therapy) is in line with a previous study including patients with traumatic PASD [15]. Part of the explanation may be that there is no evidence-based non-operative treatment for patients with traumatic PASD or RASD [17]. Nevertheless, according to the present findings, patients with PASD and RASD, not eligible for surgery, seem to have equal indications for receiving non-operative treatment.

The limitation of the current study is the small sample size, limiting the statistical power of the findings. However, the current findings are not close to being clinically relevant different why we do not believe that a larger sample size would have changed the outcome of the current study. Also, due to the requirements for the SINEX study of fulfilling the inclusion/exclusion criteria, the patients share the same eligibility criteria and may thus have had similar shoulder function. However, since the inclusion/exclusion criteria did not cover questions about the severity of the participants shoulder trouble and that no patients were excluded exclusively due to their current shoulder function we do not believe this to have affected the outcome nor the generalizability of the current findings. Further, since TSK is not validated specifically in Danish shoulder instability patients interpretation of the current TSK data needs careful interpretation. However, the fact that TSK has been validated in another Danish setting [22] and that the current population is from the same culture (Danish), using the TSK outcome instrument is not likely to bias our data. Finally, the total CMS may not reflect all aspects of shoulder function in the current population, due to its limitation in detecting shoulder instability-related problems as discussed above. However, to counter this limitation, additional measured outcomes of shoulder function were included, these being standardised CMS-ROM and CMS-strength measurements as embedded in the total CMS measurement.

The strengths of the study are the strict inclusion and exclusion criteria resulting in the homogenous study population. However, since this study is based on the eligibility criteria from an RCT [20] the current findings may be limited to patients of similar characteristics only. Though, we expect the external validity of the current study population to be high due to the multicentre design with recruitment of patients from three hospitals located in the southern, western and northern part of Denmark. Further, electronic data collection of demographic and patient reported outcomes avoided any potential assessor influence and decreased the number of missing self-reported data. Finally, with only two clinical outcome assessors, thoroughly trained in all test procedures, assessment variability was kept to a minimum for all measured outcomes.

## Conclusion

Non-operated patients with a traumatic primary anterior shoulder dislocation (PASD) and self-reported shoulder trouble three-six weeks after initial injury do not have less shoulder impairment (self-reportedly or objectively measured) than non-operated patients with recurrent (second-fifth time) anterior shoulder dislocation (RASD) and self-reported shoulder trouble three-six weeks after their latest shoulder dislocation event. Generally, both groups present with poor shoulder function and high fear of re-injury, and therefore have equal indications for receiving treatment regardless of number of previous dislocations. Since there is no evidenced non-operative supervised treatment program available, development of such program is needed.

## Abbreviations

CMS: Constant-Murley Shoulder Score
CMS-ROM: Constant-Murley Shoulder Score-Range of Motion
CMS-strength: Constant-Murley Shoulder Score-strength
EQ-VAS: EQ-5D Visual Analog Scale
GJH: Generalized Joint Hypermobility
iMVC: Isometric Maximal Voluntary Contraction
JPS: Joint Position Sense
NPRS: Numeric Pain Rating Scale
PASD: Primary Anterior Shoulder Dislocation
QoL: Quality of Life
RASD: Recurrent Anterior Shoulder Dislocation
ROM: Range of Motion
SD: Standard deviation
TSK: Tampa Scale of Kinesiophobia
WOSI: Western Ontario Shoulder Instability Index
